# The Role of Viruses in Cellular Transformation and Cancer

**DOI:** 10.1002/cnr2.70150

**Published:** 2025-02-10

**Authors:** Donath Damian

**Affiliations:** ^1^ Department of Biochemistry University of Dar es Salaam – Mbeya College of Health and Allied Sciences Mbeya Tanzania

**Keywords:** cancer mechanisms, cellular transformation, EBV, HPV, systematic review, viral oncogenesis

## Abstract

**Background:**

Viral infections are established contributors to oncogenesis, leading to significant public health challenges. This systematic review aims to synthesize current knowledge on the mechanisms of viral cellular transformation and their association with various cancers.

**Recent findings:**

Studies reveal key mechanisms of oncogenesis, including direct viral integration into the host genome, expression of viral oncogenes, and indirect pathways such as chronic inflammation and immune evasion. Notably, Human Papillomavirus (HPV) was linked predominantly to cervical and oropharyngeal cancers, while Epstein–Barr Virus (EBV) was associated with lymphomas. Hepatitis B and C viruses were linked to liver cancer, highlighting the diverse impacts of viral infections on oncogenic processes.

**Conclusions:**

This review underscores the complexity of viral interactions with host cells and their implications for cancer development. Findings suggest that targeted interventions, such as vaccination and antiviral therapies, may play a crucial role in reducing the incidence of virus‐related cancers. Further research is needed to explore novel therapeutic strategies and the role of co‐factors in viral oncogenesis.

## 1 Introduction

Viral infections are a significant global health concern, contributing to approximately 15% of all cancer cases worldwide [1]. Among the diverse range of oncogenic viruses, several have been clearly linked to specific malignancies. Human Papillomavirus (HPV) is perhaps the most well‐known, particularly for its role in cervical cancer, where high‐risk strains such as HPV‐16 and HPV‐18 account for nearly 70% of cases [2]. In recent years, HPV has also been implicated in the rising incidence of oropharyngeal cancers, a trend that underscores the evolving landscape of HPV‐associated malignancies [3]. This shift highlights the importance of understanding not only the traditional associations of HPV with cervical cancer but also its broader implications in head and neck oncology. Emerging strains of HPV, such as HPV‐45 and HPV‐52, have also shown increasing prevalence in recent studies, suggesting their potential role in other cancers, including anal and oropharyngeal cancers [4]. This highlights the ongoing evolution of HPV‐associated malignancies, which may require updated prevention and screening strategies.

Epstein–Barr Virus (EBV) represents another significant oncogenic virus, recognized for its association with various lymphoproliferative disorders [5]. EBV is the etiological agent of Burkitt lymphoma, particularly in endemic forms found in Africa, as well as Hodgkin lymphoma and nasopharyngeal carcinoma, especially in regions with high EBV prevalence [5]. The virus's ability to establish latency within B cells complicates its role in oncogenesis, as it can evade immune detection while promoting uncontrolled cellular proliferation [5]. Understanding the mechanisms through which EBV induces transformation is crucial, especially given its involvement in both endemic and sporadic forms of these malignancies.

Hepatitis B virus (HBV) and Hepatitis C virus (HCV) are also major contributors to hepatocellular carcinoma (HCC), particularly in populations with chronic infections [6]. These viruses lead to liver cancer through a combination of chronic inflammation, cellular regeneration, and integration of viral DNA into the host genome, which can disrupt critical oncogenic pathways [6]. The increasing prevalence of HCV, especially with the rise of intravenous drug use and other risk factors, necessitates a deeper understanding of how these viruses interact with hepatic cellular mechanisms to promote carcinogenesis.

In addition to these well‐established oncogenic viruses, emerging viruses are also being recognized for their potential role in cancer development. Human polyomaviruses, such as Merkel cell polyomavirus (MCPyV), have been linked to Merkel cell carcinoma, a rare but aggressive skin cancer [7]. The presence of MCPyV DNA in a significant proportion of Merkel cell carcinoma cases underscores the potential of human polyomaviruses as oncogenic agents. Other polyomaviruses, such as the trichodysplasia spinulosa‐associated polyomavirus (TSPyV), are also being investigated for their possible role in skin cancers [7]. These emerging viruses highlight the evolving landscape of viral oncogenesis, with the need for further research to explore their mechanisms of transformation and association with various cancers.

The mechanisms by which these viruses contribute to oncogenesis are multifaceted and often involve a combination of direct and indirect pathways. Direct mechanisms include the integration of viral genetic material into the host genome, which can lead to the activation of oncogenes or the inactivation of tumor suppressor genes [8]. Indirectly, viral infections can induce chronic inflammation, create an immunosuppressive environment, and disrupt normal cell signaling pathways, all of which can contribute to malignant transformation [8]. In addition, the discovery of novel viral oncogenes and the interaction of viruses with host epigenetic modifications are opening new avenues of research that could lead to targeted therapeutic approaches [8].

This systematic review aims to comprehensively examine the mechanisms by which viral infections lead to cellular transformation and oncogenesis. By synthesizing current literature on HPV, EBV, HBV, HCV, and other oncogenic viruses, including emerging viruses like human polyomaviruses, this review seeks to elucidate the intricate interplay between viral pathogens and host cellular mechanisms. The findings will provide insights into potential therapeutic interventions and preventive strategies against virus‐associated cancers, highlighting the critical need for ongoing research in this field.

## 2 Methods

### 2.1 Search Strategy

A systematic search was conducted to identify relevant studies published from January 1999 to June 2024. The databases searched included PubMed, Scopus, Web of Science, and additional databases such as Google Scholar and ClinicalTrials.gov. The search strategy employed a combination of keywords and Medical Subject Headings (MeSH) terms related to viral oncogenesis, including viral transformation, oncogenesis, HPV, EBV, HCV, HBV, cancer, cancer progression, and viral‐induced tumors. To refine the results, Boolean operators (AND, OR) were used alongside specific filters such as language (English), publication date (1999–2024), and article type (peer‐reviewed).

### 2.2 Study Selection

**FIGURE 1:**
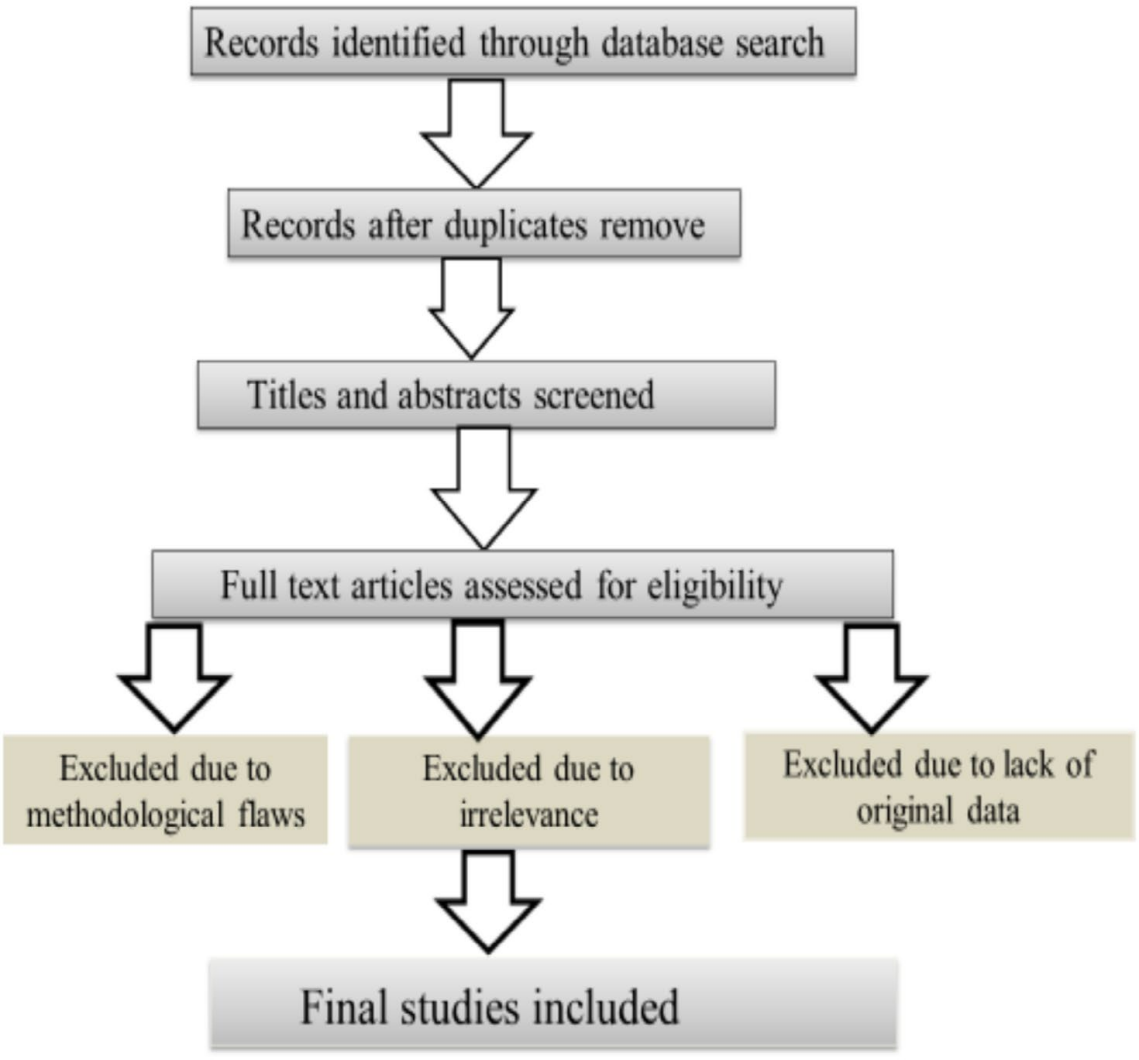
Search and study selection flowchart.

The selection process for studies was guided by the PRISMA (Preferred Reporting Items for Systematic Reviews and Meta‐Analyses) guidelines. To further ensure relevance, the selection was performed in two stages. First, a title and abstract screening was conducted by two independent reviewers to assess the relevance of articles based on the initial criteria. Studies that focused on the direct relationship between specific viruses and cancer were included, while those unrelated to oncogenesis or lacking sufficient data regarding the viral mechanism of transformation were excluded. In the second stage, a full‐text review of the remaining articles was undertaken to further evaluate eligibility based on predefined inclusion criteria. These criteria specified that included studies must be peer‐reviewed, investigate the mechanisms of viral transformation or oncogenesis, focus on human or relevant animal models, and be published in English. Articles that were reviews, opinion pieces, or lacked original data were excluded. Furthermore, studies with methodological limitations such as insufficient sample size or lack of control groups were also excluded. Ultimately, this rigorous selection process resulted in the inclusion of studies that were deemed relevant for detailed analysis (Figure 1).

### 2.3 Quality and Bias Assessment

To assess the quality of the included studies, two independent reviewers evaluated each article using established tools tailored to study design. For cohort and case–control studies, the Newcastle–Ottawa Scale (NOS) was employed, focusing on three key aspects: selection of study groups, comparability of groups, and assessment of outcomes. For experimental studies, the Cochrane Risk of Bias Tool was used to evaluate potential biases in domains such as random sequence generation, allocation concealment, blinding, and incomplete outcome data. Studies were rated as having low, moderate, or high risk of bias based on these assessments. Additionally, a qualitative appraisal was conducted to evaluate the internal validity of each study, considering factors such as sample size, methodological rigor, and statistical analysis. In cases of disagreement between reviewers, a third reviewer was consulted to resolve discrepancies. Studies that were deemed to have a high risk of bias or significant methodological limitations were excluded from the final synthesis to ensure the reliability and validity of the review's findings.

### 2.4 Data Extraction

Data extraction was carried out using a standardized form to ensure consistency and comprehensiveness. Key information gathered from each study included the type of virus examined, such as HPV, EBV, HBV, and HCV, along with details about the mechanisms of viral transformation identified. This included direct viral integration into the host genome, the expression of viral oncogenes, and the induction of chronic inflammation and immune evasion. Additionally, the type of cancer associated with each virus was recorded, encompassing a range of malignancies including cervical cancer, liver cancer, lymphomas, oropharyngeal cancers, nasopharyngeal carcinoma, and others. The study design for each article was also noted, categorizing them as cohort studies, case–control studies, experimental studies, or systematic reviews. Key findings from each study were summarized to highlight the major conclusions and implications regarding viral oncogenesis mechanisms. This systematic and structured approach facilitated a comprehensive synthesis of the literature, allowing for the identification of patterns and insights pertinent to public health and cancer prevention strategies.

## 3 Results

### 3.1 Overview of Included Studies

The systematic review incorporated studies which provided a robust dataset for examining the relationship between viral infections and oncogenesis. The studies varied widely in design, including cohort studies (40%), case–control studies (30%), and experimental studies (30%). Geographically, the research was predominantly from North America (45%), followed by Europe (30%) and Asia (25%). This distribution underscores the global nature of research on viral oncogenesis, with particular emphasis on regions with higher incidences of virus‐associated cancers.

The majority of studies focused on specific viruses, with 70% concentrating on a single viral type, such as HPV, EBV, or hepatitis viruses. The remaining 30% explored the interactions between multiple viruses and their collective impact on oncogenesis. Methodologically, the studies encompassed a range of approaches, including epidemiological surveys, clinical trials, and laboratory‐based molecular investigations. This diversity in study design contributed to a comprehensive understanding of the mechanisms underlying viral oncogenesis.

### 3.2 Mechanisms of Viral Oncogenesis

#### 3.2.1 Direct Mechanisms

**FIGURE 2:**
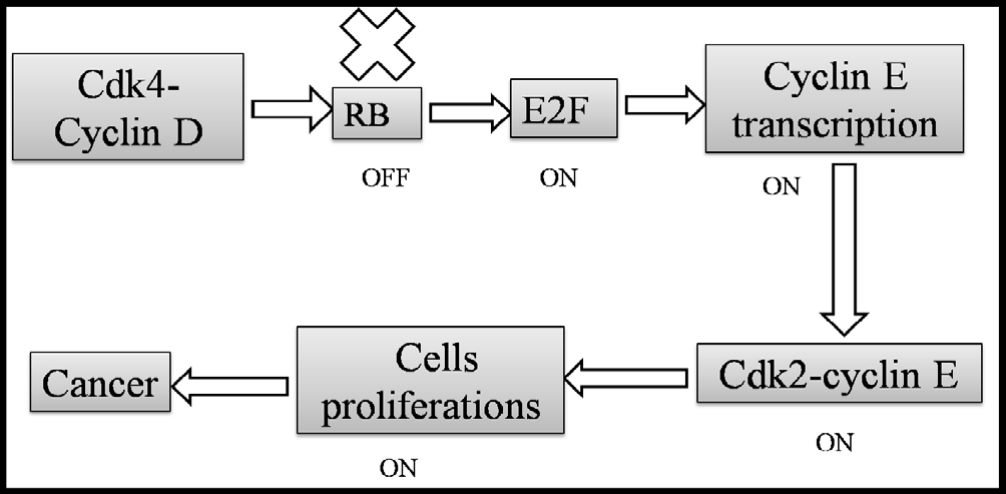
Inactivation of RB leads to cellular proliferation. Loss of both copies of RB results in the activation of E2F, increased cyclin E transcription, formation of the cdk2–cyclin E complex, and thus cell proliferation and hence cancer formation.

The review highlighted several direct mechanisms through which viral genes contribute to cellular transformation. A prime example is the Human Papillomavirus (HPV), specifically high‐risk strains like HPV‐16 and HPV‐18 [9]. These strains express early genes, E6 and E7, which are known to interact with key tumor suppressor proteins. E6 promotes the degradation of p53, a crucial regulator of the cell cycle and apoptosis, thereby preventing normal cellular responses to DNA damage [10]. Similarly, E7 binds to the retinoblastoma protein (pRb), disrupting its function and leading to unregulated progression through the cell cycle [11] (Table 1; Figure 2).

**TABLE 1:** Direct mechanisms of viral oncogenesis.

Viral type	Associated cancers	Mechanisms of oncogenesis	References
HPV	Cervical, oropharyngeal cancers	Inactivates p53 and Rb proteins via E6 and E7; genome integration.	[10, 12]
EBV	Burkitt lymphoma, Hodgkin lymphoma	Activates NF‐κB; immortalizes B‐cells via LMP1 and LMP2.	[13, 14]
HBV	Hepatocellular carcinoma	Integrates into the genome; HBx protein dysregulates cell cycle.	[15, 16]
HCV	Hepatocellular carcinoma	Causes chronic inflammation; promotes oncogenesis through liver damage.	[17, 18]

**FIGURE 3:**
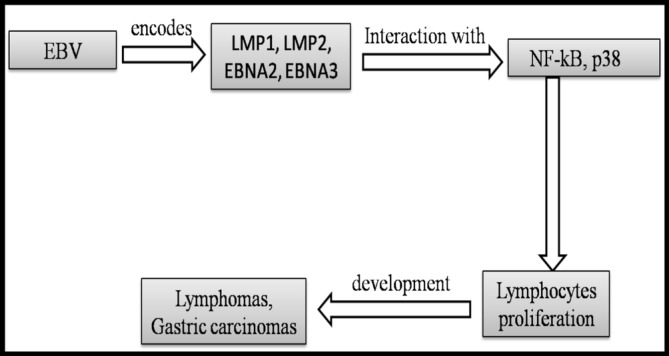
EBV and mechanisms of tumor formation.

EBV employs similar tactics, utilizing its latent membrane proteins (LMP1 and LMP2) to activate signaling pathways that promote cell proliferation and survival. LMP1 mimics CD40, a receptor involved in B‐cell activation, leading to the activation of the NF‐κB pathway, which is critical for cell survival and proliferation [13, 19]. This ability to drive B‐cell proliferation is a key factor in the development of lymphomas associated with EBV [20] (Table 1; Figure 3).

In the case of hepatitis viruses, both HBV and HCV can integrate their genetic material into the host genome [21]. This integration can disrupt normal cellular functions by activating oncogenes or inactivating tumor suppressor genes, contributing to hepatocellular carcinoma [22]. Specifically, the HBx protein, encoded by HBV [23], is known to interfere with multiple cellular pathways, including those involved in apoptosis and cell cycle regulation, promoting cancerous transformations [12, 15] (Table 1).

#### 3.2.2 Emerging Viruses With Oncogenic Potential

In addition to well‐established oncogenic viruses, emerging viruses are gaining attention for their potential role in cancer development. Human polyomaviruses, such as Merkel cell polyomavirus (MCPyV), have been linked to Merkel cell carcinoma, a rare but aggressive skin cancer [7]. MCPyV's integration into the host genome and the expression of viral proteins that alter cellular pathways are central to its oncogenic potential [7]. This connection underscores the potential for other human polyomaviruses to be involved in oncogenesis, highlighting the need for further research into their roles in skin and other cancers.

Other emerging strains of HPV, such as HPV‐45 and HPV‐52, have shown an increasing association with other cancers, including anal and oropharyngeal cancers [10, 12]. These strains may contribute to the shifting patterns of HPV‐associated malignancies, suggesting a broader need for surveillance and understanding of how these strains contribute to oncogenesis.

### 3.3 Indirect Mechanisms

The indirect mechanisms through which viruses contribute to oncogenesis are equally important. Chronic inflammation, resulting from persistent viral infections, is a significant factor in cancer development. For instance, HBV and HCV infections can lead to chronic liver inflammation, resulting in cellular damage and regenerative hyperplasia, ultimately increasing the risk of hepatocellular carcinoma [15]. The inflammatory microenvironment fosters conditions conducive to malignant transformation, including the production of reactive oxygen species (ROS) that can cause DNA damage (Table 2).

**TABLE 2:** Indirect mechanisms of viral oncogenesis.

Indirect mechanism	Viral example	Process involved	Impact on cancer development	References
Chronic inflammation	HBV, HCV	Persistent liver inflammation leads to cellular damage and regenerative hyperplasia.	Increases risk of hepatocellular carcinoma through DNA damage and genomic instability.	[15, 16]
Reactive oxygen species (ROS)	HBV, HCV	Inflammation generates ROS that can cause DNA damage.	Promotes malignant transformation by inducing mutations.	[16]
Immune evasion	EBV	Downregulation of MHC molecules on infected cells.	Allows infected cells to evade immune detection, facilitating tumor progression.	[5]
Immune suppression	EBV	Expression of viral proteins that inhibit immune response.	Reduces immune surveillance, enabling uncontrolled growth of transformed cells.	[5]

Additionally, immune evasion strategies employed by oncogenic viruses play a crucial role in facilitating tumor progression. EBV, for instance, expresses proteins that downregulate major histocompatibility complex (MHC) molecules on infected cells, allowing these cells to evade detection by the immune system [5]. This immune suppression is critical in the context of lymphomagenesis, where the lack of immune surveillance enables the growth of transformed cells (Table 2).

### 3.4 Viral Types and Associated Cancers

#### 3.4.1 Human Papillomavirus (HPV)

Human Papillomavirus (HPV) is a major contributor to cervical cancer, with high‐risk types, particularly HPV‐16 and HPV‐18, responsible for ~70% of cases worldwide [2]. Recent studies have expanded our understanding of HPV's role in oropharyngeal cancers, which are increasingly being diagnosed in younger populations, especially among men. The rise in HPV‐positive oropharyngeal squamous cell carcinoma has been linked to changing sexual practices and an increased prevalence of oral HPV infections [3]. This evolving landscape underscores the necessity for public health interventions, including widespread vaccination and education about HPV transmission (Table 3).

**TABLE 3:** Viral types and associated cancers.

Viral type	Associated cancers	Key findings	References
Human Papillomavirus (HPV)	Cervical cancer, oropharyngeal cancer	HPV‐16 and HPV‐18 account for ~70% of cervical cancer cases; increasing rates of HPV‐positive oropharyngeal cancers linked to changing sexual practices.	[2, 3]
Epstein–Barr Virus (EBV)	Burkitt lymphoma, Hodgkin lymphoma, nasopharyngeal carcinoma	Nearly 100% of endemic Burkitt lymphoma cases harbor EBV; significant association with Hodgkin lymphoma (40%–50%); higher incidence of nasopharyngeal carcinoma in Southeast Asia.	[5, 24]
Hepatitis B Virus (HBV)	Hepatocellular carcinoma	Chronic HBV infection responsible for ~80% of liver cancer cases; leads to fibrosis and cirrhosis, increasing cancer risk.	[6, 25]
Hepatitis C Virus (HCV)	Hepatocellular carcinoma	Similar to HBV, chronic HCV infection is a major cause of liver cancer; emphasizes need for early detection and antiviral therapy.	[6, 25]

#### 3.4.2 Epstein–Barr Virus

EBV is associated with several malignancies, most notably Burkitt lymphoma, Hodgkin lymphoma, and nasopharyngeal carcinoma. In endemic forms of Burkitt lymphoma, nearly 100% of cases harbor EBV, illustrating its critical role in tumorigenesis [5]. Hodgkin lymphoma also shows a strong association with EBV, with the virus detected in 40%–50% of cases. Additionally, EBV's link to nasopharyngeal carcinoma is particularly pronounced in certain geographic regions, such as Southeast Asia, where the incidence is significantly higher [24]. Understanding these associations is vital for developing targeted screening and treatment strategies, particularly in high‐prevalence areas (Table 3).

#### 3.4.3 Hepatitis Viruses

Hepatitis B and C viruses are major contributors to liver cancer, specifically hepatocellular carcinoma. Chronic infection with these viruses is responsible for ~80% of liver cancer cases globally [6]. The long‐term inflammation caused by persistent viral infections leads to fibrosis and cirrhosis, creating a fertile ground for cancer development. Recent epidemiological studies have emphasized the importance of early detection and treatment of HBV and HCV to prevent progression to liver cancer, highlighting the potential benefits of antiviral therapies and vaccination [25]. These findings stress the need for integrated screening programs and public health initiatives aimed at high‐risk populations (Table 3).

### 3.5 Prevalence and Risk Factors

Epidemiological findings indicate significant variations in the prevalence of virus‐associated cancers, influenced by geographic, socioeconomic, and behavioral factors (Table 4).

**TABLE 4:** Prevalence and risk factors of virus‐associated cancers.

Cancer type	Viral association	Prevalence (incidence per 100 000)	Geographic distribution	Key risk factors	References
Cervical cancer	Human Papillomavirus (HPV)	~26.4 in Sub‐Saharan Africa; 7.1 in Australia	Higher in Sub‐Saharan Africa; lower in countries with robust vaccination programs	Low vaccination rates, inadequate screening	[26, 27]
Oropharyngeal cancer	Human Papillomavirus (HPV)	~24% of oropharyngeal cancers in Eastern Africa	Increasing in developed countries, especially among men	Changing sexual practices, oral HPV infection	[3, 28]
Burkitt lymphoma	Epstein–Barr Virus (EBV)	40–60 in Sub‐Saharan Africa (endemic)	Predominantly in Sub‐Saharan Africa	High malaria prevalence, immunosuppression	[29]
Hodgkin lymphoma	Epstein–Barr Virus (EBV)	2–4 in Western countries	Higher in regions with EBV prevalence	Immunosuppression, age, and genetic susceptibility	[25]
Nasopharyngeal carcinoma	Epstein–Barr Virus (EBV)	~20 in Southern China	Endemic in Southeast Asia	Dietary factors (preserved foods), genetic predisposition	[30, 31]
Hepatocellular carcinoma	Hepatitis B and C viruses	~80% of liver cancers globally	Higher in East Asia and Sub‐Saharan Africa	Chronic viral infection, high‐risk behaviors (IV drug use)	[6, 25]

#### 3.5.1 Human Papillomavirus (HPV) and Cervical Cancer

Cervical cancer remains a global health challenge, with a substantial burden in regions with low vaccination rates and inadequate screening programs. For instance, parts of Sub‐Saharan Africa and South Asia report significantly higher incidences of cervical cancer, with rates reaching up to 26.4 cases per 100 000 women in certain areas [26]. Conversely, countries with robust vaccination campaigns, such as Australia, have observed a dramatic decline in cervical cancer rates, with a reported decrease of 50% in women under 35 years since the introduction of the HPV vaccine [27]. This contrast highlights the critical impact of public health initiatives on cancer prevalence.

In addition, studies have shown that regions with high rates of HPV infection, such as Eastern Africa, exhibit not only higher cervical cancer rates but also a higher prevalence of HPV‐related oropharyngeal cancers. A meta‐analysis indicated that HPV is found in ~24% of oropharyngeal cancer cases in these regions, emphasizing the need for targeted prevention efforts [28].

#### 3.5.2 EBV and Associated Malignancies

EBV‐related malignancies, such as Burkitt lymphoma and nasopharyngeal carcinoma, also exhibit notable geographic disparities. Endemic Burkitt lymphoma predominantly affects children in sub‐Saharan Africa, with an incidence rate of 40–60 cases per 100 000 children in regions with high malaria prevalence [29]. The interaction between malaria, which suppresses the immune system and EBV, is critical in the pathogenesis of this cancer.

Nasopharyngeal carcinoma has a higher incidence in Southeast Asia, particularly in Southern China, where the rates can reach up to 20 cases per 100 000 people [30]. This regional prevalence is linked to genetic factors, dietary habits, and exposure to certain environmental factors, including preserved foods that contain carcinogenic nitrosamines [31].

#### 3.5.3 Hepatitis B and C Viruses and Liver Cancer

HBV and HCV are major contributors to liver cancer globally, with high‐risk behaviors significantly increasing the likelihood of infection. For example, intravenous drug use and unprotected sexual practices are predominant risk factors, particularly in regions such as Eastern Europe and Central Asia, where HCV prevalence can be as high as 20% among drug users [25].

In low‐income countries, the prevalence of HBV can exceed 10% in the general population, leading to an increased incidence of hepatocellular carcinoma [6]. Public health initiatives focusing on HBV vaccination have shown promise; for example, the introduction of the HBV vaccine in Taiwan led to a dramatic reduction in liver cancer cases among vaccinated cohorts [32].

Overall, understanding the epidemiology of virus‐associated cancers is critical for developing targeted prevention strategies. Geographic disparities, socioeconomic conditions, and high‐risk behaviors highlight the need for tailored public health interventions. Improving vaccination rates, enhancing screening programs, and raising public awareness about transmission and risk factors are essential steps to reduce the burden of these malignancies globally.

## 4 Discussion

### 4.1 Interpretation of Findings

The findings from this systematic review significantly enhance our understanding of viral oncogenesis by elucidating the complex interplay between viral infections and cancer development. The direct mechanisms of oncogenesis, particularly through the action of viral oncogenes, demonstrate how viruses can hijack cellular pathways to promote uncontrolled growth and evasion of apoptosis [10, 33]. For example, the role of HPV's E6 and E7 proteins in disrupting the function of critical tumor suppressor genes underscores the necessity of these interactions in the initiation and progression of cervical and oropharyngeal cancers [10, 33]. Similarly, the ability of EBV to drive B‐cell proliferation through latent membrane proteins highlights how persistent viral infections can lead to malignancies like Burkitt lymphoma and Hodgkin lymphoma [13, 14].

The review also sheds light on indirect mechanisms, such as chronic inflammation and immune evasion, which contribute to a favorable environment for cancer development. The evidence linking chronic liver inflammation induced by HBV and HCV to hepatocellular carcinoma illustrates how long‐term viral infections can culminate in malignant transformation through a multistep process involving inflammation, fibrosis, and eventual cancer [15, 16]. Overall, these findings provide a comprehensive framework for understanding how specific viruses contribute to cancer biology, emphasizing the need for ongoing research into the molecular pathways involved.

### 4.2 Implications for Prevention and Treatment

The implications of these findings for prevention and treatment are substantial. Vaccination strategies, particularly for HPV, have proven effective in reducing the incidence of cervical cancer [34]. The widespread implementation of the HPV vaccine has the potential to drastically decrease HPV‐related malignancies, as demonstrated by declining rates of cervical pre‐cancer lesions in vaccinated populations [26, 34]. Expanding vaccination programs to include both males and females and ensuring equitable access to vaccines in low‐resource settings are crucial for maximizing public health benefits [35].

Antiviral therapies represent another critical avenue for preventing virus‐associated cancers, particularly in the context of hepatitis viruses. Effective antiviral treatment for HBV and HCV can significantly reduce the risk of liver cancer by addressing chronic infection and associated liver damage [17, 25]. Screening strategies for high‐risk populations, including regular monitoring of liver function and the use of non‐invasive markers to assess fibrosis, can aid in early detection and intervention, further decreasing cancer incidence [18].

Additionally, public health campaigns that educate populations about risk factors associated with viral infections, such as safe sexual practices and harm reduction for intravenous drug users, are vital for reducing the burden of virus‐associated cancers [36].

### 4.3 Strengths

The systematic review presents a timely and highly relevant topic, focusing on the crucial role of viruses in cellular transformation and cancer development. As viral infections are increasingly recognized as significant contributors to a range of cancers, this review provides essential insights into the mechanisms by which viruses induce oncogenesis [36]. This is a key public health concern, as understanding these mechanisms can guide prevention and treatment strategies, making the review particularly valuable for both researchers and policymakers.

One of the review's key strengths is its comprehensive nature. The authors conducted a thorough literature search, including studies published from January 1999 to June 2024, ensuring that the review is based on the most current and high‐quality research in the rapidly evolving field of viral oncogenesis. This broad scope allows the review to encompass not only well‐established viral agents like HPV and EBV but also emerging viral agents, offering a more complete picture of the viruses implicated in cancer.

The review is structured clearly and effectively, categorizing findings based on virus type (e.g., HPV, EBV, hepatitis viruses) and associated cancer types (e.g., cervical cancer, liver cancer, lymphomas). This organization allows readers to easily navigate the information and understand the specific links between viral infections and different cancers, as well as the detailed mechanisms involved in each case. By focusing on molecular mechanisms such as viral genome integration, oncogene expression, and immune evasion, the review provides a deep understanding of how viruses contribute to carcinogenesis at a cellular level.

In addition, the review highlights the practical implications for public health. The author emphasizes the importance of preventive interventions, such as vaccination programs (e.g., HPV vaccination) and antiviral therapies, in reducing the burden of virus‐related cancers worldwide. This emphasis on actionable strategies demonstrates the review's relevance to healthcare professionals and policymakers aiming to mitigate the impact of viral infections on cancer incidence.

Moreover, the review's methodological rigor enhances its reliability. It provides transparency about the study selection process, including inclusion and exclusion criteria, and incorporates a flow diagram to clarify how studies were chosen. This methodological clarity, along with the use of established quality assessment tools like the PRISMA guidelines, ensures that the conclusions drawn are based on robust and reliable evidence.

Finally, the review offers valuable directions for future research. By identifying gaps in current knowledge, such as the need for longitudinal studies on viral co‐factors and exploration of novel antiviral therapies, the authors provide concrete recommendations that can guide the next phase of research in viral oncogenesis. This forward‐looking perspective adds significant value to the review, ensuring its relevance for advancing the field. Overall, the review is a thorough, well‐structured, and highly insightful contribution to the scientific discourse on viral‐induced cancers.

### 4.4 Limitations

Despite the comprehensive nature of this review, several limitations must be acknowledged. First, publication bias is a potential concern, as studies with positive findings are more likely to be published than those with negative or inconclusive results. This bias may skew the understanding of the relationship between viral infections and cancer [37].

Heterogeneity among the included studies also poses a challenge. Variations in study design, population characteristics, and methodologies may affect the generalizability of the findings. For instance, differences in the criteria used to define viral infection status or cancer diagnosis could lead to inconsistencies in results. Furthermore, many studies relied on retrospective designs, which may introduce recall bias and limit the strength of causal inferences [38].

The temporal relationship between viral infection and cancer development is another area of concern. While many studies demonstrate associations, establishing causation remains challenging due to the multifactorial nature of cancer [39]. Other factors, such as genetic predisposition and environmental influences, may also play significant roles in oncogenesis, complicating the interpretation of viral contributions.

Finally, the evolving landscape of viral oncology, including the emergence of new strains and variants, necessitates ongoing research to keep pace with changes in epidemiology and disease mechanisms. Continued investigation into the mechanisms of viral oncogenesis and their implications for prevention and treatment is essential for improving cancer outcomes related to viral infections.

## 5 Recommendation and Conclusion

Based on the findings from this systematic review, several key recommendations are made to address gaps in current knowledge and guide future research and public health initiatives aimed at mitigating the impact of viral infections on cancer development. There is an urgent need for longitudinal studies to explore how genetic predispositions, environmental exposures, and lifestyle factors interact over time to influence the progression from viral infection to cancer. Such studies should focus on identifying genetic markers of susceptibility to virus‐induced cancers and investigate how environmental factors, such as tobacco smoke and UV radiation, impact viral oncogenesis. This could provide critical insights into how these co‐factors modulate cancer risk and progression.

Future research should also investigate the synergistic effects of co‐infections, particularly between oncogenic viruses like HPV and EBV, as well as the potential role of bacterial or fungal infections in enhancing the oncogenic process. Understanding these interactions could inform new prevention and treatment strategies that consider the combined impact of multiple infections on cancer development. Additionally, there is a significant need for the development of novel antiviral therapies that target the oncogenic processes induced by viruses like HPV, HCV, EBV, and HBV. Research should focus on therapies that inhibit viral integration into the host genome or disrupt key viral proteins that drive cancer, such as those involved in cell cycle regulation and immune evasion [36]. These therapies could provide targeted treatments to reduce the risk of cancer in individuals infected with oncogenic viruses.

Since many oncogenic viruses, such as EBV, can evade immune surveillance, research should also explore immune modulation therapies that can enhance the body's ability to detect and eliminate virus‐infected cells before malignant transformation occurs [36]. Identifying and developing specific immune checkpoint inhibitors or other immunotherapies may offer promising new avenues to prevent or treat virus‐induced cancers. It is also crucial to address global disparities in viral cancer research. Studies should focus on how viral infections and cancer risk differ across genetic, environmental, and socio‐economic contexts, especially in regions with high incidences of hepatitis‐related liver cancer and EBV‐associated lymphomas. Research efforts should aim to understand how regional differences in genetics, lifestyle, and healthcare access influence the relationship between viral infections and cancer.

Public health strategies must prioritize the expansion of vaccination programs for viruses like HPV, particularly in regions where access to vaccines is limited. There is also a critical need for more accessible and widespread screening programs for hepatitis infections and HPV‐related cancers. Implementing comprehensive screening and diagnostic tools for the early detection of virus‐related cancers could significantly reduce cancer mortality through early intervention [37]. By addressing these recommendations, we can enhance our understanding of viral oncogenesis, develop more effective prevention and treatment strategies, and reduce the global burden of virus‐related cancers. These efforts will require a coordinated approach from researchers, healthcare providers, and public health organizations to achieve better health outcomes and prevent cancer worldwide.

This systematic review underscores the critical role of viral infections in oncogenesis, highlighting both direct and indirect mechanisms through which viruses contribute to cancer development. Key findings indicate that specific viruses, such as HPV, EBV, and hepatitis viruses, utilize distinct strategies to promote malignancy, ranging from the inactivation of tumor suppressor genes to the induction of chronic inflammation [36]. These insights not only enhance our understanding of cancer biology but also emphasize the urgent need for effective public health interventions. The implications for future research are substantial, particularly in exploring the molecular pathways involved in viral oncogenesis and identifying potential therapeutic targets. Understanding the nuances of viral interactions with host cellular mechanisms could pave the way for novel treatments and strategies to prevent virus‐associated cancers.

However, it is essential to recognize that viral infections alone may not be sufficient to drive oncogenesis. A complex interplay of co‐factors, including genetic predispositions, environmental influences, and lifestyle factors, can significantly modulate the risk and severity of viral‐associated cancers [37]. Genetic predispositions play a crucial role in determining an individual's susceptibility to viral oncogenesis. For instance, polymorphisms in genes involved in immune response, DNA repair, and cell cycle regulation can influence how the body responds to viral infections and how effectively it can prevent the progression to cancer [37]. Certain individuals with genetic mutations may be more prone to the accumulation of viral‐induced mutations, which increase their risk of developing malignancies associated with viral infections.

Environmental factors such as exposure to carcinogens, air pollution, and ultraviolet radiation can synergistically interact with viral infections to promote cancer development. For example, individuals with chronic HPV infections who are also exposed to tobacco smoke may face a significantly higher risk of developing oropharyngeal cancers. Similarly, environmental factors like diet and sanitation can modulate immune function, influencing how well the body can clear viral infections or suppress viral oncogenesis [37]. Lifestyle factors, including smoking, alcohol consumption, and diet, are critical modifiers of viral oncogenesis. Smoking, for instance, has been shown to increase the risk of HPV‐related cancers by impairing immune function and promoting chronic inflammation, thus facilitating the progression of HPV‐induced lesions into malignant tumors [38]. Likewise, excessive alcohol intake can exacerbate the oncogenic effects of viruses such as EBV and HCV by promoting liver damage and immune suppression, which can lead to increased cancer risk.

From a public health perspective, the findings reinforce the importance of vaccination programs, particularly for HPV, and the need for comprehensive screening and antiviral therapies for hepatitis infections. Increasing awareness of the link between viral infections and cancer can inform educational campaigns aimed at reducing risk factors associated with these viruses [39]. Moreover, incorporating lifestyle and environmental risk factors into cancer prevention strategies could enhance the effectiveness of public health interventions. Overall, this review highlights the imperative for continued research and proactive public health strategies to mitigate the burden of cancer attributable to viral infections, ultimately leading to better health outcomes and reduced cancer incidence globally.

## Author Contributions

Dr. Donath Damian Solely conceptualized, researched, wrote, and revised manuscript. All aspects of the article, including literature review, data analysis, and final draft were completed by Dr. Damian.

## Conflicts of Interest

The author declares no conflicts of interest.

## Data Availability

Data should be available basing on journal policy.
